# Stay or stray? Evidence for alternative mating strategy phenotypes in both men and women

**DOI:** 10.1098/rsbl.2014.0977

**Published:** 2015-02

**Authors:** Rafael Wlodarski, John Manning, R. I. M. Dunbar

**Affiliations:** 1 Department of Experimental Psychology, University of Oxford, Oxford OX1 3UD, UK; 2 Department of Psychology, Northumbria University, Newcastle-upon-Tyne, UK

**Keywords:** mating strategies, 2D : 4D, sociosexuality, monogamy, promiscuity

## Abstract

In all comparative analyses, humans always fall on the borderline between obligate monogamy and polygamy. Here, we use behavioural indices (sociosexuality) and anatomical indices (prenatal testosterone exposure indexed by 2D : 4D digit ratio) from three human populations to show that this may be because there are two distinct phenotypes in both sexes. While males are more promiscuous and display higher prenatal testosterone exposure than females overall, our analyses also suggest that the within-sex variation of these variables is best described by two underlying mixture models, suggesting the presence of two phenotypes with a monogamous/promiscuous ratio that slightly favours monogamy in females and promiscuity in males. The presence of two phenotypes implies that mating strategy might be under complex frequency-dependent selection.

## 1. Introduction

Whenever comparative analyses of mammalian mating systems are undertaken, humans invariably fall midway between monogamous and polygamous species [1,2]. Although no explanation has ever been offered for this, one plausible explanation is that humans actually consist of a mix of short-term (promiscuous) and long-term (monogamous) mating phenotypes. The extent to which any one individual pursues a short-term mating strategy (‘unrestricted’ strategy involving promiscuous mating with multiple partners) or a long-term mating strategy (‘restricted’ strategy favouring the formation of exclusive and extended pair-bonds) has been referred to as their ‘sociosexual orientation’ [3].

Cross-cultural research widely confirms that males, the lesser-investing sex, are typically more sociosexually ‘unrestricted’ (promiscuous) than females [4]. While long-term mating strategies that involve increased parental investment may enhance a male's chances of offspring survival [5], they do so at the expense of lost mating opportunities [6,7]. Although these two strategies could well just be opposite ends of the same continuum, it has sometimes been assumed (albeit without any real evidence) that these represent two distinct male phenotypes: those that pursue a more promiscuous, unrestricted mating strategy (‘stray’) and those that focus on investing more heavily in their offspring in long-term relationships (‘stay’) [8–11]. Although individual differences in female mating strategies have sometimes been noted in the literature [10,12], the possibility that women might also exhibit contrasting mating strategies has received considerably less attention.

We tested the hypothesis that there are distinct mating strategy phenotypes in both men and women using two large datasets: a North American and British sample of 595 individuals who completed the sociosexual orientation inventory (SOI-R) [13] and a British sample of 1314 individuals whose 2D : 4D digit ratios were measured. The SOI-R indexes an individual's psychological degree of sexual promiscuity on a continuum running from restricted (monogamous) to unrestricted (promiscuous). The 2D : 4D ratio is an anatomical marker for fetal testosterone exposure and testosterone receptor-site density [14,15], and reflects the level of prenatal testosterone effects in the adult phenotype [16]. Across primates, 2D : 4D ratio correlates with mating system [2,17] and provides a biological marker for mating strategy. To test the hypothesis, we first determined whether or not the distribution of each index for each sex was most likely to be unimodal or bimodal, with the latter being indicative of a mixture of two underlying distributions. Where the latter was the case, we determined the mean and variance for each distribution.

## 2. Methods

SOI-R data were collected from British and North American Caucasian participants using an online questionnaire [18]. These comprised 134 male and 186 female British participants and 68 male and 187 female North American participants (ages 18–63, mean = 24.7, s.d. = 7.9). To assess participants' preferred mating strategy (sociosexual orientation), questions forming the ‘attitude’ and ‘desire’ subscales of the SOI-R were used [3,13].

Data on 2D : 4D ratios were previously collected from right-hand photocopies in a large-scale study of a British Caucasian population (*n* = 1314, 572 males, 742 females).

Statistical analyses were carried out with R and the *mixtools* package for finite mixture model analysis using model-based clustering (version 0.4.6) [19]. This analysis assumes underlying Gaussian distributions of any modelled modes—an assumption that seems reasonable as most genetic and behavioural human variation is typically normally distributed. To determine whether each dataset was a mixture of two distributions, we first used a likelihood ratio test (asymptomatic *χ*^2^) to compare multicomponent distributions with a single-mode model. Maximum-likelihood estimation (MLE) was then used to iteratively estimate multimodal Gaussian distributions that maximized model fit to the observed data, varying component mixing proportions (*λ*), means (*μ*) and standard deviations (*σ*). Mean best-fit model parameter estimates and standard errors were further estimated using parametric bootstrapping (1000 realizations).

## 3. Results

**Figure 1.**
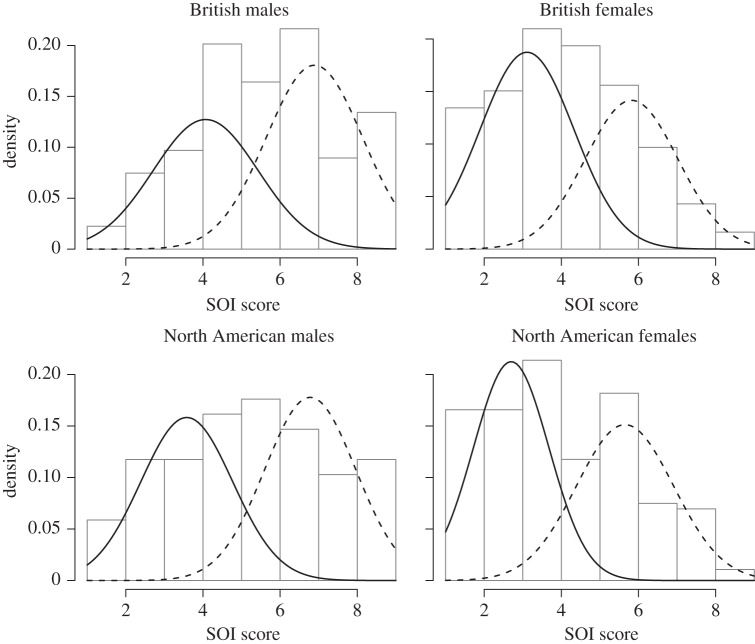
Modelled within-sex distribution mixtures of sociosexuality in British and North American samples, plotted against a histogram of the data. Curves display best-fit models estimating underlying mixture distributions: solid lines represent low-sociosexuality (restricted or monogamous) phenotype, dashed line high-sociosexuality (unrestricted or promiscuous) phenotype.

The likelihood ratio *χ*^2^ tests confirm that the British and North American male and female sociosexuality datasets each have an underlying bimodal distribution (table 1). Modelling confirmed the existence of two phenotypes within each sex, one of low (restricted) sociosexuality and the other of high (unrestricted) sociosexuality. High-sociosexuality males make up a slightly larger proportion of the male distribution in each case, and low-sociosexuality females make up a slightly larger proportion of the female distributions (table 1). Figure 1 shows the modelled phenotype distributions overlying a histogram for the observed data.

**Table 1.** Modelled distribution estimates for sociosexuality in British and North American samples.

	restricted sociosexuality (bootstrap mean ± s.e.)	unrestricted sociosexuality (bootstrap mean ± s.e.)
British males		
* test for bimodality:* (*χ*^2^(1, *n* = 134) = 4*.*88, *p* = 0*.*027)		
mixing proportion (*λ*)	0.434 ± 0.245	0.566 ± 0.245
mean (*μ*)	4.07 ± 0.89	6.89 ± 0.63
variance (*σ*)	1.36 ± 0.39	1.25 ± 0.34
British females		
*test for bimodality:* (*χ*^2^(1, *n* = 186) = 15*.*08, *p* < 0*.*001)		
mixing proportion (*λ*)	0.573 ± 0.262	0.426 ± 0.262
mean (*μ*)	3.11 ± 0.68	5.82 ± 0.98
variance (*σ*)	1.22 ± 0.34	1.20 ± 0.40
North American males		
*test for bimodality:* (*χ*^2^(1, *n* = 68) = 8*.*87, *p* = 0*.*003)		
mixing proportion (*λ*)	0.473 ± 0.232	0.527 ± 0.232
mean (*μ*)	3.58 ± 0.89	6.78 ± 0.75
variance (*σ*)	1.19 ± 0.44	1.18 ± 0.40
North American females		
*test for bimodality:* (*χ*^2^(1, *n* = 187) = 9*.*75, *p* = 0*.*002)		
mixing proportion (*λ*)	0.522 ± 0.124	0.478 ± 0.124
mean (*μ*)	2.70 ± 0.28	5.65 ± 0.45
variance (*σ*)	0.98 ± 0.17	1.26 ± 0.24

**Figure 2.**
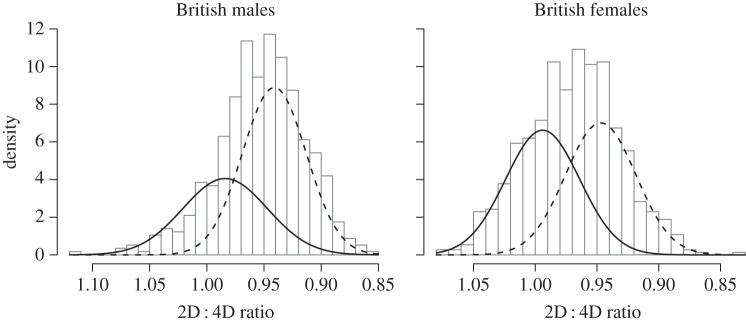
Modelled within-sex distribution mixtures of 2D : 4D ratio (reversed *x*-axis) in a British sample, plotted against a histogram of the data. Curves display best-fit models estimating underlying mixture distributions: solid lines representing low-testosterone (high-2D : 4D ratio) phenotype, dashed lines high-testosterone (low-2D : 4D ratio) phenotype.

While the *χ*^2^ tests confirm that the male 2D : 4D data also have an underlying bimodal distribution, the female data just fail to reach statistical significance (*p* = 0.079). Nonetheless, modelling still supports the existence of two underlying phenotypes for both sexes (table 2), with low 2D : 4D males making up a larger proportion of the male distribution, and the female 2D : 4D phenotypes being more evenly distributed (figure 2).

**Table 2.** Modelled distribution estimates for 2D : 4D ratio in British sample.

	low testosterone (bootstrap mean ± s.e.)	high testosterone (bootstrap mean ± s.e.)
British males		
*test for bimodality:* (*χ*^2^(1, *n* = 572) = 15*.*17, *p* < 0*.*001)		
mixing proportion (*λ*)	0.376 ± 0.235	0.624 ± 0.235
mean (*μ*)	0.984 ± 0.027	0.941 ± 0.006
variance (*σ*)	0.037 ± 0.009	0.028 ± 0.005
British females		
*test for bimodality:* (*χ*^2^(1, *n* = 742) = 3*.*07, *p* = 0*.*079)		
mixing proportion (*λ*)	0.498 ± 0.326	0.501 ± 0.326
mean (*μ*)	0.994 ± 0.023	0.947 ± 0.020
variance (*σ*)	0.030 ± 0.008	0.0285 ± 0.009

## 4. Discussion

This study is the first, to the best of knowledge, to show statistically that both men and women exhibit two reproductive phenotypes of varying proportions. This would seem to provide a principled explanation for the fact that humans always appear midway between monogamous and polygamous species on all anatomical indices of mating system. Overall, our results suggest that the proportional split in males slightly favours an unrestricted (short-term) mating strategy, with a 57 : 43 split on average for the three datasets, whereas females have a reversed split (47 : 53). However, the mixing proportions in the 2D : 4D digit ratio dataset suggest that a slightly higher proportion of the unrestricted phenotype is present in both sexes (males approx. 62%, females approx. 50%). Note that although males are, overall, more unrestricted than females in all three datasets (as has widely been reported to be the case [4,20]), there is in fact considerable overlap: unrestricted females have more extreme (i.e. more promiscuous) indices than restricted males in each sample. This would not have been predicted on conventional views of human mating style. Of course, while the limitations of the available statistical tools have obliged us to approach the data in the way we have, our analysis does not formally allow us to determine whether the phenotypes we identify represent two separate subpopulations, each with their own normal distribution, or a single population with two modes. Deciding between these, and other, options will require further biological study.

While it has been widely suggested that males divide into two mating types (‘cads’ versus ‘dads’ [8]) and there is some evidence for a genetic basis for this distinction [11], this study is the first, to the best of our knowledge, to provide quantitative evidence on their proportional distributions in natural populations. More importantly, it is the first to suggest that a similar partition may also exist in females. Although the genetic variation underlying sociosexual behaviour in a female twin population had been previously found on visual inspection to be clearly bimodally distributed [10], quantitative evidence for distinct phenotypes underlying such bimodality has been lacking.

The statistical method used here assumes that underlying mixture distributions are normally distributed and does not rule out the possibility that the two phenotypes are skewed or represent two separate peaks on a single underlying distribution. Unfortunately, it is not possible to investigate alternative statistical distributions: the near-infinite number of possible permutations and combinations involved makes statistical analysis impossible. These methods, however, are still robust enough to point to the existence of alternative mating phenotypes in both sexes, and it is likely that these have distributions across the phenotypic continuum (in all likelihood reflecting the fact that they are predispositions rather than categorical types). More importantly, which of these alternatives is biologically the case does not affect our claim that statistical analysis of three separate datasets reveals that each sex seems to exhibit two different phenotypes in roughly equal proportions.

There has been some debate concerning the distinction between trait and type views of personality dimensions [21], with some evidence to suggest that what have previously been seen as types (e.g. extrovert versus introvert) are in fact part of a trait continuum where differential binning of data can create the illusion of two underlying mixture distributions. Our analyses are inevitably subjected to the same risk, of course. However, we rest our claim not on the way we cast the data as a histogram or, as in the case of personality types, on particular theoretical preconceptions, but rather on a purely statistical method based on a quantitative approach to the data that we use simply to demonstrate that the data are not best described by a unimodal normal distribution. Exactly what this means in terms of the underlying biology we leave to future research to discover.

Accepting our analyses as offering at least *prima facie* evidence for the existence of distinct mating phenotypes in the two sexes prompts a number of predictions for future investigation. If the two phenotypes essentially represent stable and unstable pair-bonding predispositions (see Walum *et al*. [11]), we might expect there to be some tendency for assortative mating between the phenotypes. We might also predict that stable–stable pairings are less likely to divorce than other pairings, with unstable–unstable pairings having the shortest durations. The existence of two phenotypes raises a number of further evolutionary questions. One is whether there are within-sex fitness differences between the two strategies. There is some evidence to suggest that reproductive success is linearly related to 2D : 4D ratios, but in opposite directions in the two sexes [14], but whether this is enough to drive the evolution of such a pattern has yet to be determined. While it is possible that the four-way division is an evolutionary stable strategy (ESS) and in evolutionary balance, it is also possible that the distribution is inherently unstable, because the two sexes are in conflict over the optimal balance between mating and parental investment.

Finally, we noted above that 2D : 4D ratios (a biological marker) are slightly more biased towards a promiscuous (unrestricted) strategy than the SOI index (a psychological behavioural index). While the magnitude of the difference is small in each case, the discrepancy suggests that mating strategy inclinations might also be subjected to a modest degree of cultural modification. Previous research has found that female sociosexuality is more responsive to environmental shifts than male sociosexuality [4,22], and our data confirm this: while both sexes exhibit a shift (towards a restricted strategy in males, but towards unrestricted in females), the magnitude of the shift is larger in women than in men. While there is strong evidence that additive genetic factors best predict adult sociosexuality [23], differences in behaviour are in part likely to reflect cultural or environmental fine tuning of underlying genetic strategies in response to local circumstances as each sex tries to maximize overall fitness.

## Supplementary Material

DATA_Manning_White_UK_2D4D.xlsx

## Supplementary Material

DATA_SOI_Desire_Attitude.xlsx
